# Supplementary material for: dcHiC detects differential compartments across multiple Hi-C datasets
Source: Nat Commun. 2022 Nov 11;13:6827. doi: 10.1038/s41467-022-34626-6 (PMC9652325; doi:10.1038/s41467-022-34626-6)
Supplement: Supplementary file 1 — Supplementary Information [file 41467_2022_34626_MOESM1_ESM.pdf]

Supplementary Figure S1: Inconsistent A/B compartment assignments between *dcHiC* and HOMER. **(A-B)** Shows the number (top) and fraction (bottom) of consistent and inconsistent A/B assignments by *dcHiC* and HOMER in mouse ESC and NPC Hi-C maps. **(C)** Shows the Lamin B1 signal of inconsistently labelled regions with dcHiC-B but HOMER-A regions showing lamin B1 association supporting their heterochromatin (or B) assignment (n=821, 307, 1415, and 144 respectively). **(D)** Shows the expression distribution of genes overlapping with the inconsistently labelled regions by *dcHiC* and HOMER (n=1220, 180, 1851, and 147 respectively). Data are presented as mean values ± SEM. Source data are provided as a Source Data file.

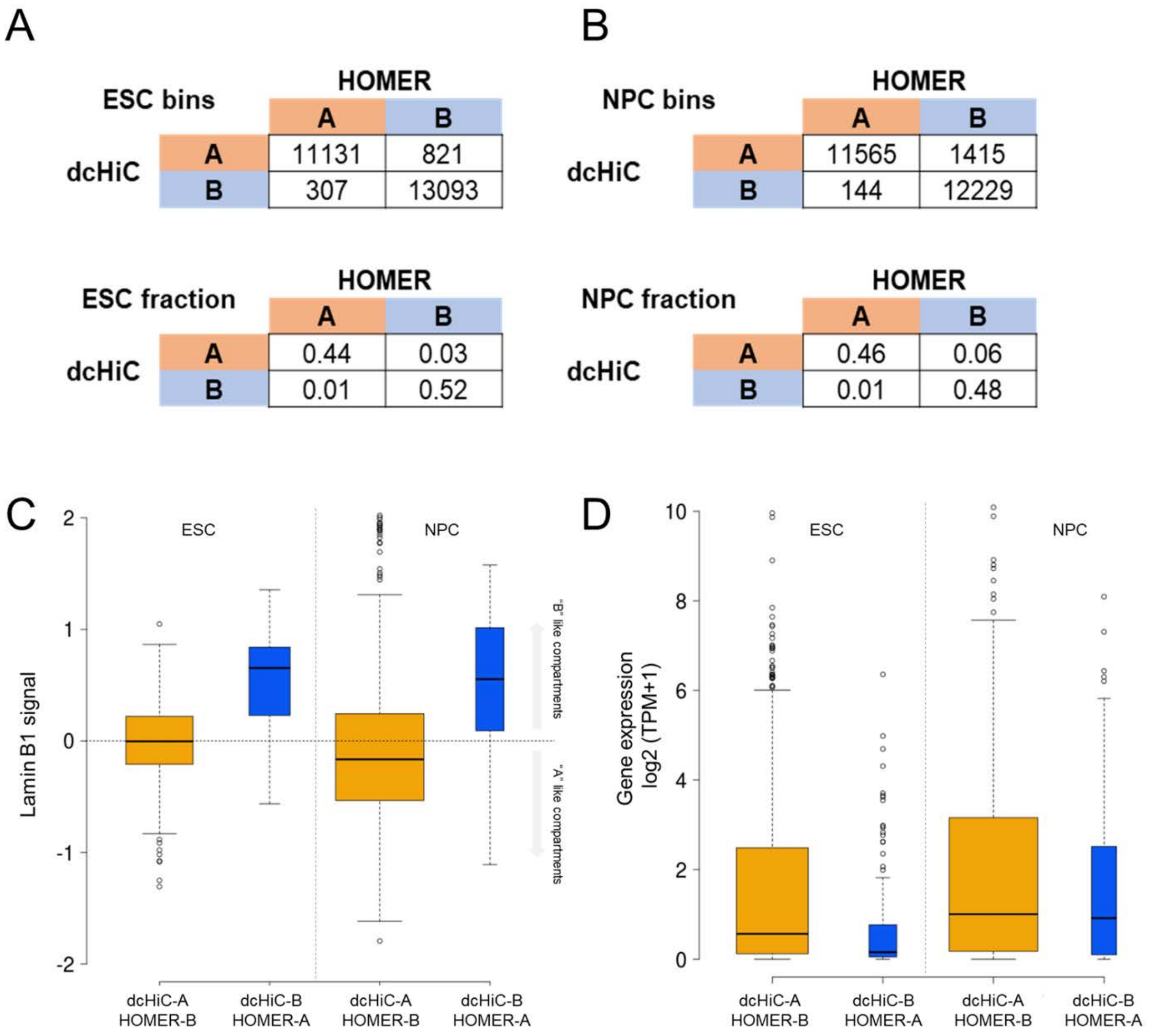

Supplementary Table S1: The table shows the relative time uses of HOMER as compared to dcHiC. Across the four different resolutions (100Kb to 20Kb), on average dcHiC is ~22 times faster than HOMER in calculating the genome-wide compartment scores. HOMER did not finish (DNF) the compartment calculation at 10Kb resolution. Source data are provided as a Source Data file.

Total run time ratio (HOMER/dcHiC)					
Down sampling rates (100% = 500 million)	ESC resolution (Replicate 1 and 2)				
	100Kb	50Kb	40Kb	20Kb	10Kb
100%	14.05	24.62	22.17	33.30	DNF
80%	15.39	19.73	25.62	37.96	DNF
60%	18.18	21.17	15.05	37.77	DNF
40%	11.87	16.71	16.56	52.29	DNF
20%	11.52	12.62	13.49	45.77	DNF
10%	14.41	9.79	11.48	45.22	DNF

Supplementary Table S2: The table shows the relative time uses of CscoreTool as compared to dcHiC. Across the five different resolutions (100Kb to 10Kb), on average dcHiC is ~7 times faster than CscoreTool in calculating the genome-wide compartment scores. Source data are provided as a Source Data file.

Total run time ratio (CscoreTool/dcHiC)					
Down sampling rates (100% = 500 million)	ESC resolution (Replicate 1 and 2)				
	100Kb	50Kb	40Kb	20Kb	10Kb
100%	8.63	9.42	9.54	13.49	4.75
80%	10.02	8.26	11.06	13.13	4.66
60%	8.23	9.51	7.39	12.74	4.22
40%	4.93	8.09	7.55	15.39	3.73
20%	2.91	4.77	5.10	10.65	2.23
10%	2.62	2.70	3.60	6.97	1.32

Supplementary Table S3: The table shows the relative memory consumption of HOMER as compared to dcHiC. Across the four different resolutions (100Kb to 20Kb), on average dcHiC is consuming ~1.4 times less memory than HOMER in calculating the genome-wide compartment scores. HOMER did not finish (DNF) the compartment calculation at 10Kb resolution.

Memory consumption ratio (HOMER/dcHiC)					
Down sampling rates (100% = 500 million)	ESC resolution (Replicate 1 and 2)				
	100Kb	50Kb	40Kb	20Kb	10Kb
100%	3.64	1.44	1.02	0.83	DNF
80%	3.99	1.23	1.01	1.03	DNF
60%	2.23	1.01	0.85	1.26	DNF
40%	1.92	0.76	0.92	1.53	DNF
20%	1.22	0.58	0.74	2.23	DNF
10%	0.56	1.07	1.20	3.07	DNF

Supplementary Table S4: The table shows the relative memory consumption of CscoreTool as compared to dcHiC. Across the five different resolutions (100Kb to 10Kb), on average CscoreTool is using ~1.2 times less memory than dcHiC in calculating the genome-wide compartment scores.

Memory consumption ratio (CscoreTool/dcHiC)					
Down sampling rates (100% = 500 million)	ESC resolution (Replicate 1 and 2)				
	100Kb	50Kb	40Kb	20Kb	10Kb
100%	0.71	0.84	0.79	1.02	0.70
80%	0.95	0.83	0.89	1.17	0.83
60%	0.68	0.81	0.81	1.28	0.65
40%	0.95	0.75	1.12	1.12	0.52
20%	0.84	0.77	0.78	0.89	0.30
10%	0.62	1.06	0.74	0.67	0.15

Supplementary Figure S2: Comparison of **(A)** absolute log2Fold change of gene expression difference and **(B)** -log10(adj.P-value) of this difference from DESeq2 for all the genes overlapping with dcHiC and HOMER exclusive differential compartments detected from comparing mouse ESC to NPC (n=675, 1585 for both). Data are presented as mean values ± SEM. Source data are provided as a Source Data file.

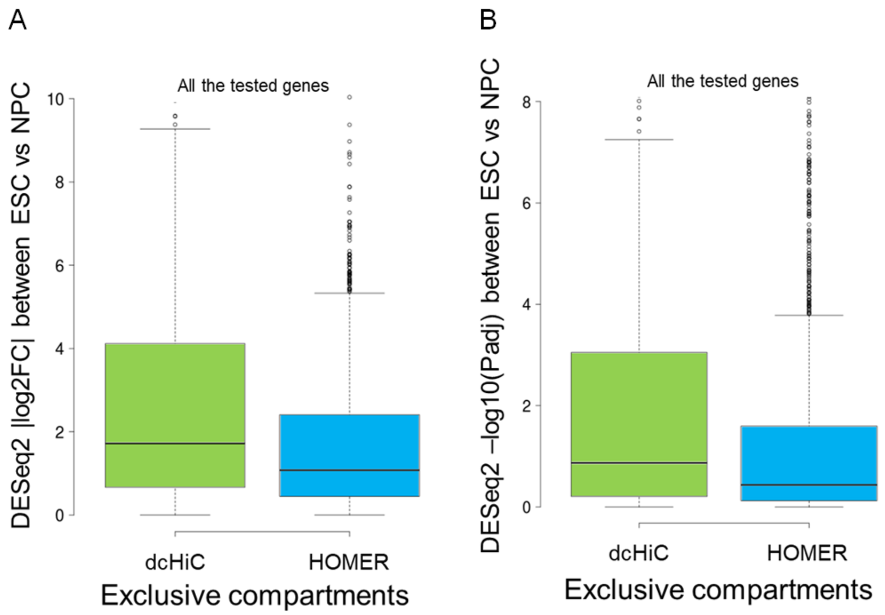

Supplementary Figure S3: Average difference in absolute number of histone peaks (MACS2 p-value < 1e-5) per 100Kb of differential bins within *dcHiC* and HOMER differential compartments. **(A)** Shows the absolute difference of average number H3K27ac peaks between ESC and NPC per 100Kb of dcHiC and HOMER differential compartments. The result shows that there is more difference in H3K27ac peaks per 100Kb of differential compartments identified by *dcHiC*. **(B-C)** The result shows that are more differences in both H3K4me3 and H3K4me1 peaks per 100Kb of differential compartments called by *dcHiC* compared to HOMER. Source data are provided as a Source Data file.

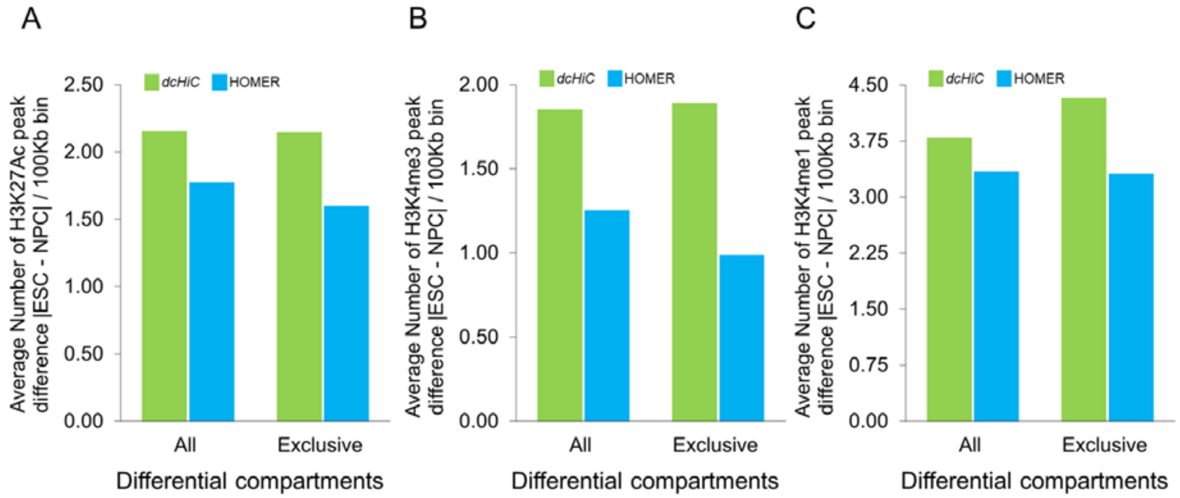

Supplementary Figure S4: Effect of sequencing depth on pairwise differential compartment calls (mouse ESC vs NPC) by *dcHiC*. The panel shows the percentages of differential compartments detected at 100% sequencing depth that are recovered after down-sampling the biological replicates (4 for ESC and 3 for NPC) at different levels (80% to 10%) for 100kb resolution analysis. Source data are provided as a Source Data file.

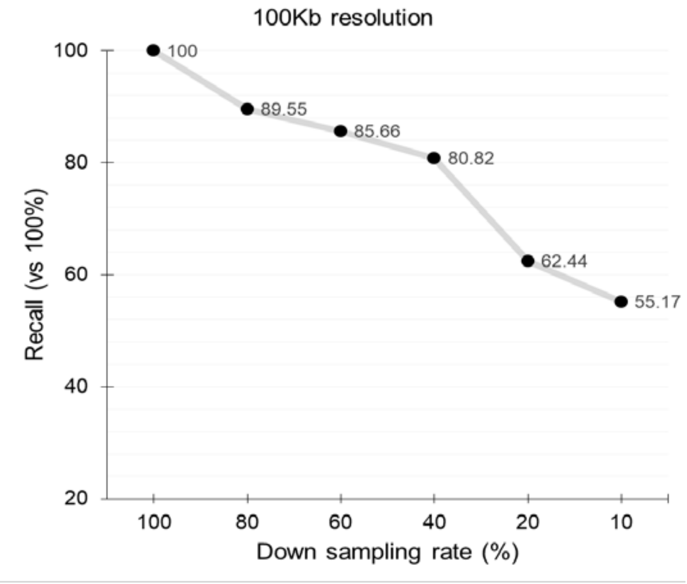

Supplementary Figure S5: Effect of sequencing depth on pairwise differential compartment calls (mouse ESC vs NPC) by *dcHiC*. **(A-D)** The plots showing recovery percentages of differential compartments detected at 100% sequencing depth after down-sampling (similar to Supplementary Figure S4) for four additional resolutions: 50Kb, 40Kb, 25Kb and 10Kb. Source data are provided as a Source Data file.

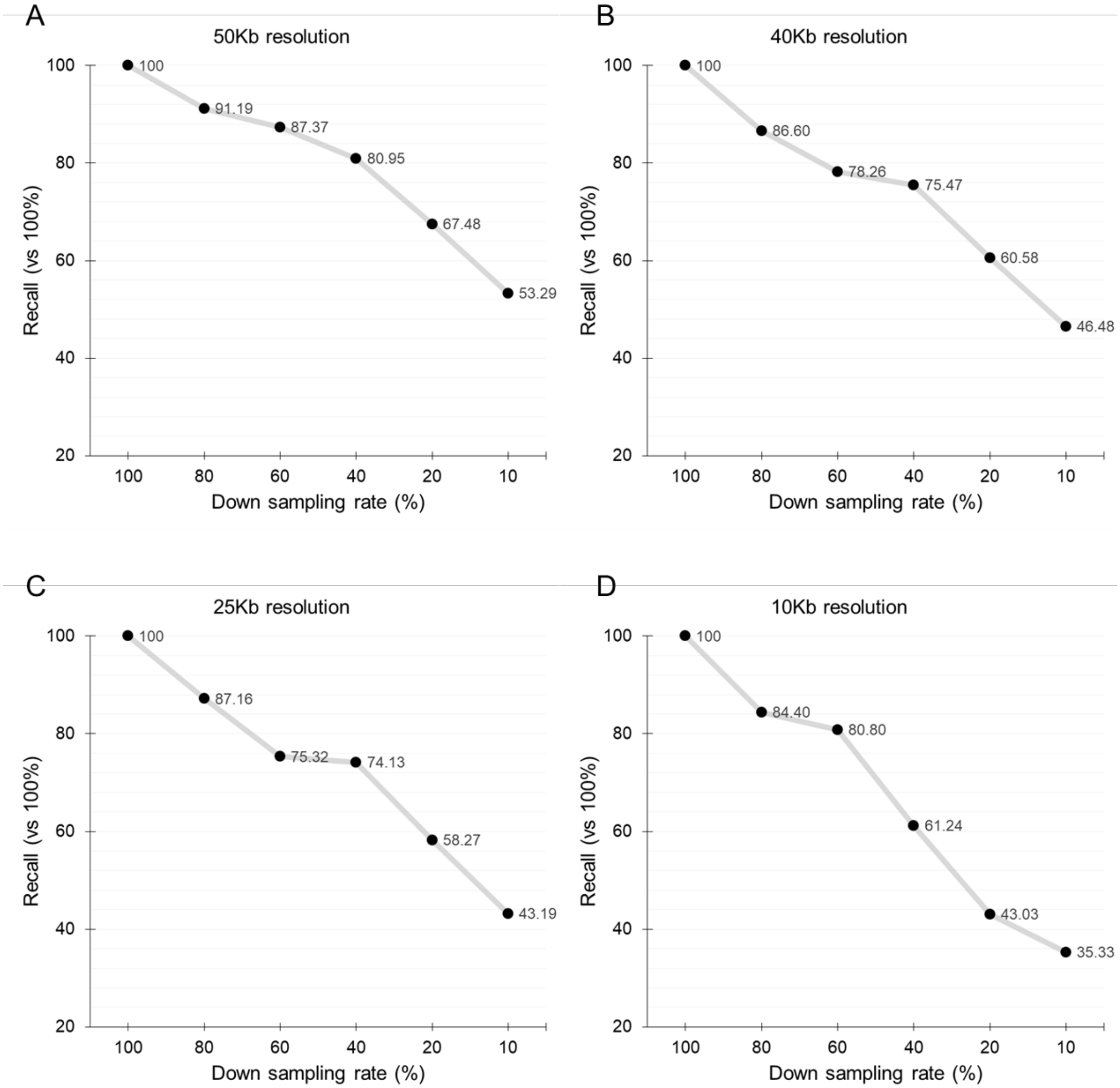

Supplementary Figure S6: Comparison of 100Kb and 10Kb dcHiC analysis of ESC – NPC compartments. **(A)** Genomic region containing Dppa2/4 genes with 10kb vs 100kb differences at the boundaries of common differential compartments. **(B)** A chromosome 13 region with differential compartments that are identified only at the 10Kb resolution *dcHiC* analysis. Source data are provided as a Source Data file.

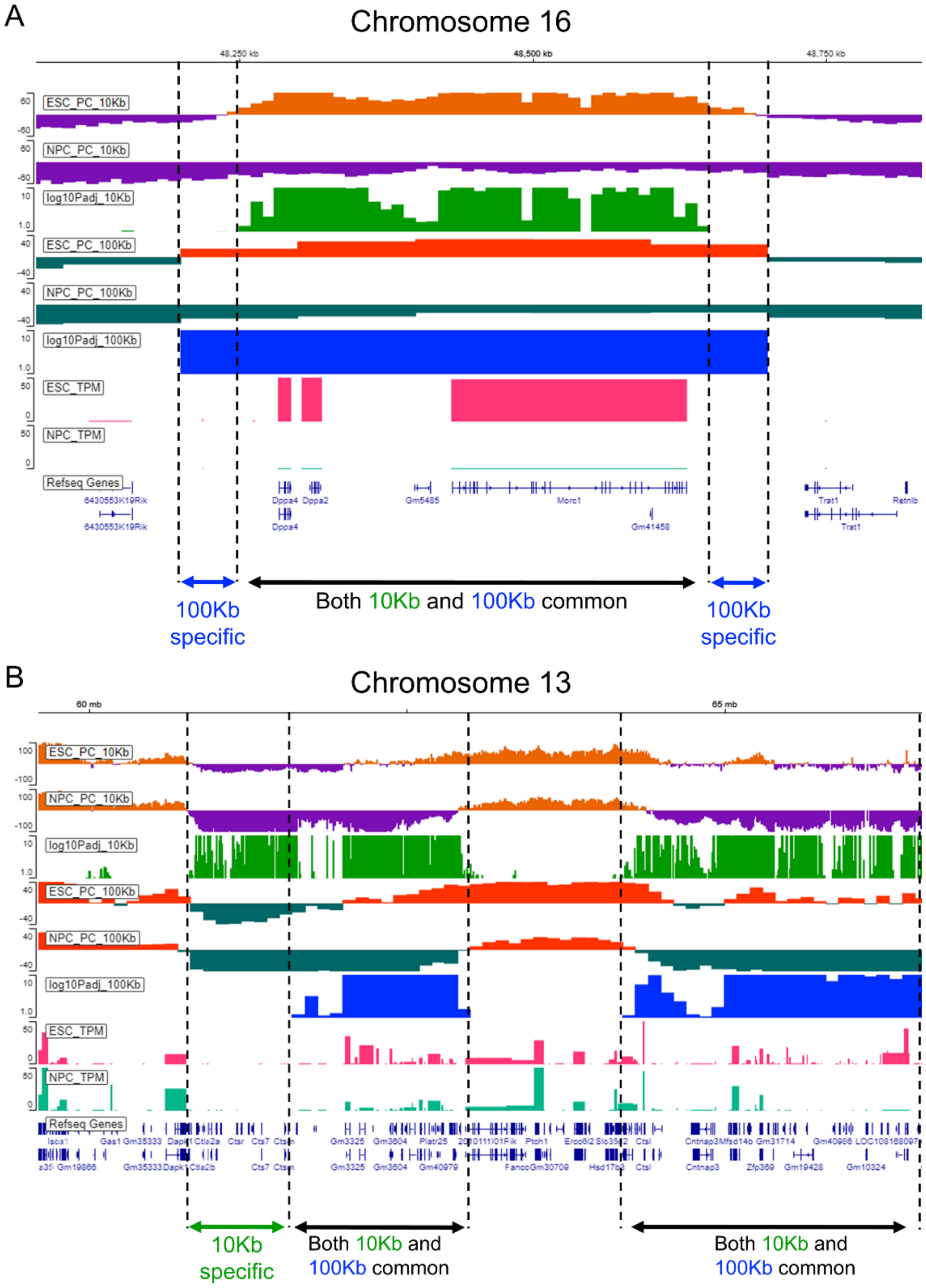

Supplementary Table S5: Effect of resolution and sequencing depth on type-1 error rate. The table shows differential compartments identified by comparing two pseudo-replicates against each other using *dcHiC* on mouse ESC. The comparison is repeated at different sequencing depths (rows) and resolutions (columns).

Down sampling rates (100% = 500 million)	ESC (Pseudo-replicate 1 vs 2)				
	100Kb	50Kb	40Kb	25Kb	10Kb
100%	0	0	0	0	0
80%	0	0	0	0	0
60%	0	0	0	0	0
40%	0	0	0	0	0
20%	0	0	0	0	0
10%	0	0	0	2	6

Supplementary Table S6: Effect of down-sampling on the correlation of compartment scores between two mouse ESC pseudo-replicates.

Compartment correlations	Down sampling rates (100% = 500 million)	ESC Pseudo-replicate 2					
		100%	80%	60%	40%	20%	10%
ESC Pseudo-replicate 1	100%	1.00	0.99	0.95	0.97	0.82	0.76
	80%	0.99	1.00	0.96	0.96	0.81	0.74
	60%	0.95	0.96	1.00	0.90	0.75	0.69
	40%	0.97	0.96	0.90	1.00	0.83	0.77
	20%	0.82	0.81	0.75	0.83	1.00	0.78
	10%	0.76	0.74	0.69	0.77	0.78	1.00

Supplementary Table S7: Effect of down-sampling on the correlation of compartment scores between two mouse ESC pseudo-replicates after removing certain chromosomes.

Compartment correlations (Excluding chr4, chr5, chr14, chr17, chrX)	Down sampling rates (100% = 500 million)	ESC Pseudo-replicate 2					
		100%	80%	60%	40%	20%	10%
ESC Pseudo-replicate 1	100%	1.00	1.00	0.97	0.98	0.87	0.85
	80%	1.00	1.00	0.94	0.96	0.86	0.84
	60%	0.97	0.94	1.00	0.88	0.78	0.76
	40%	0.98	0.96	0.88	1.00	0.86	0.84
	20%	0.87	0.86	0.78	0.86	1.00	0.78
	10%	0.85	0.84	0.76	0.84	0.78	1.00

Supplementary Table S8: Effect of differential down-sampling (different down-sampling rates for each replicate) on the differential compartment detection by *dcHiC* between two mouse ESC pseudo-replicates.

Differential compartments	Down sampling rates (100% = 500 million)	ESC Pseudo-replicate 2					
		100%	80%	60%	40%	20%	10%
ESC Pseudo-replicate 1	100%	0	0	0	0	247	827
	80%	0	0	0	84	353	907
	60%	0	0	0	124	348	1215
	40%	0	84	124	0	335	1027
	20%	247	353	348	335	0	737
	10%	827	907	1215	1027	737	0

Supplementary Table S9: Effect of differential down-sampling (different down-sampling rates for each replicate) on the differential compartment detection by *dcHiC* between two mouse ESC pseudo-replicates after removing certain chromosomes.

Differential compartments (Excluding chr4, chr5, chr14, chr17, chrX)	Down sampling rates (100% = 500 million)	ESC Pseudo-replicate 2					
		100%	80%	60%	40%	20%	10%
ESC Pseudo-replicate 1	100%	0	0	0	0	3	170
	80%	0	0	0	0	20	205
	60%	0	0	0	2	22	467
	40%	0	0	2	0	25	292
	20%	3	20	22	25	0	469
	10%	170	205	467	292	469	0

Supplementary Figure S7: Differential compartments are associated with sub-compartment transitions during ESC to NPC lineage differentiation. **(A-B)** shows the total number of differential compartment transitions, grouped based on their sub-compartment classes within ESC and NPC lineages. **(C-D)** Shows the background normalized transitions, or the fold-change values obtained from differential sub-compartment frequencies divided by non-differential sub-compartment changes. **(E)** Sub-compartment flipping corresponds to changes in genomic activity: he panel shows a significant alteration in the gene expression pattern when ESC-A.2.1 flips to NPC-B.2.2 sub-compartment (n=149 genes). (F) One such flip in chromosome 4 (145-148Mb region) encompassing 71 unique genes. The average expression of these genes in ESC is around 20 TPM and ~0.4 TPM in NPC. Data are presented as mean values ± SEM. Source data are provided as a Source Data file.

dcHiC differential compartments overlapping with Calder sub-compartment transitions during ESC to NPC lineage differentiation

A

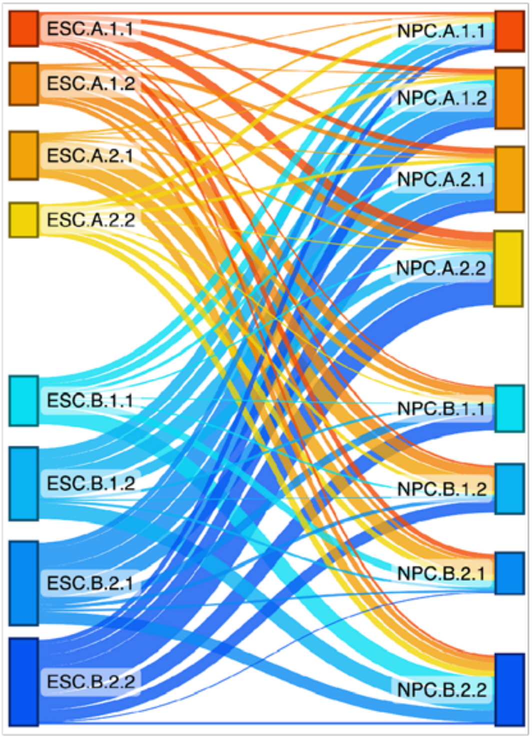

C

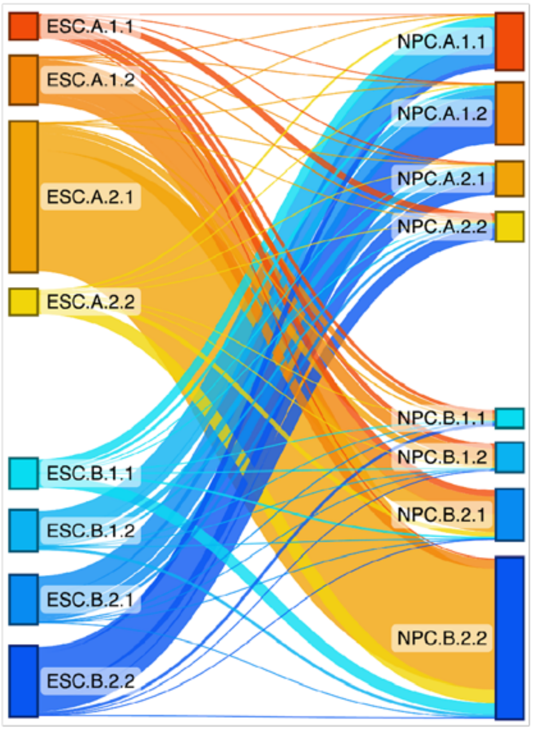

B

From ESC \ To NPC	A.1.1	A.1.2	A.2.1	A.2.2	B.1.1	B.1.2	B.2.1	B.2.2
A.1.1	9	21	23	39	7	10	22	10
A.1.2	3	3	20	31	35	41	15	21
A.2.1	10	4	3	10	22	58	50	37
A.2.2	20	20	16	5	7	15	25	27
B.1.1	31	32	14	8	3	9	31	74
B.1.2	44	49	73	45	12	3	11	68
B.2.1	24	80	71	62	34	16	10	53
B.2.2	21	44	53	115	69	51	3	10

D

From ESC \ To NPC	A.1.1	A.1.2	A.2.1	A.2.2	B.1.1	B.1.2	B.2.1	B.2.2
A.1.1	0.03	0.24	1.44	6.01	3.16	4.69	6.09	1.35
A.1.2	0.05	0.05	0.45	1.56	5.20	11.10	16.61	8.53
A.2.1	0.65	0.10	0.10	0.56	1.60	6.37	16.46	112.7
A.2.2	2.06	0.85	0.63	0.24	0.39	1.17	4.35	12.65
B.1.1	8.21	2.91	0.96	0.29	0.14	0.50	2.13	10.86
B.1.2	19.14	6.22	5.52	2.18	0.48	0.11	0.31	2.84
B.2.1	16.24	13.73	8.56	2.86	1.39	0.35	0.14	0.68
B.2.2	4.92	31.53	12.42	11.58	2.91	1.03	0.03	0.04

E

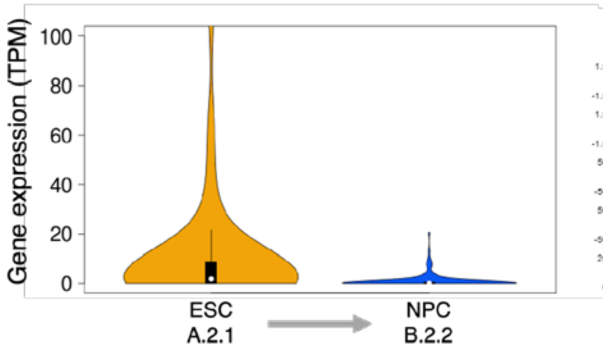

F

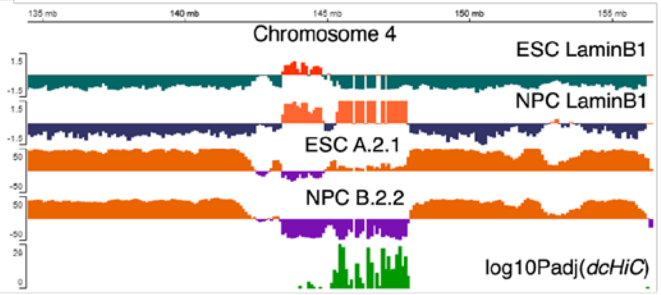

Supplementary Figure S8: Time-series clustering of mouse Long-term hematopoietic stem-cells (LT-HSC) to Granulocyte (GR) differentiation based on differential compartment scores. **(A)** Shows the six cluster patterns obtained. **(B)** Functional enrichment results based on the genes overlapping regions within each cluster. Source data are provided as a Source Data file.

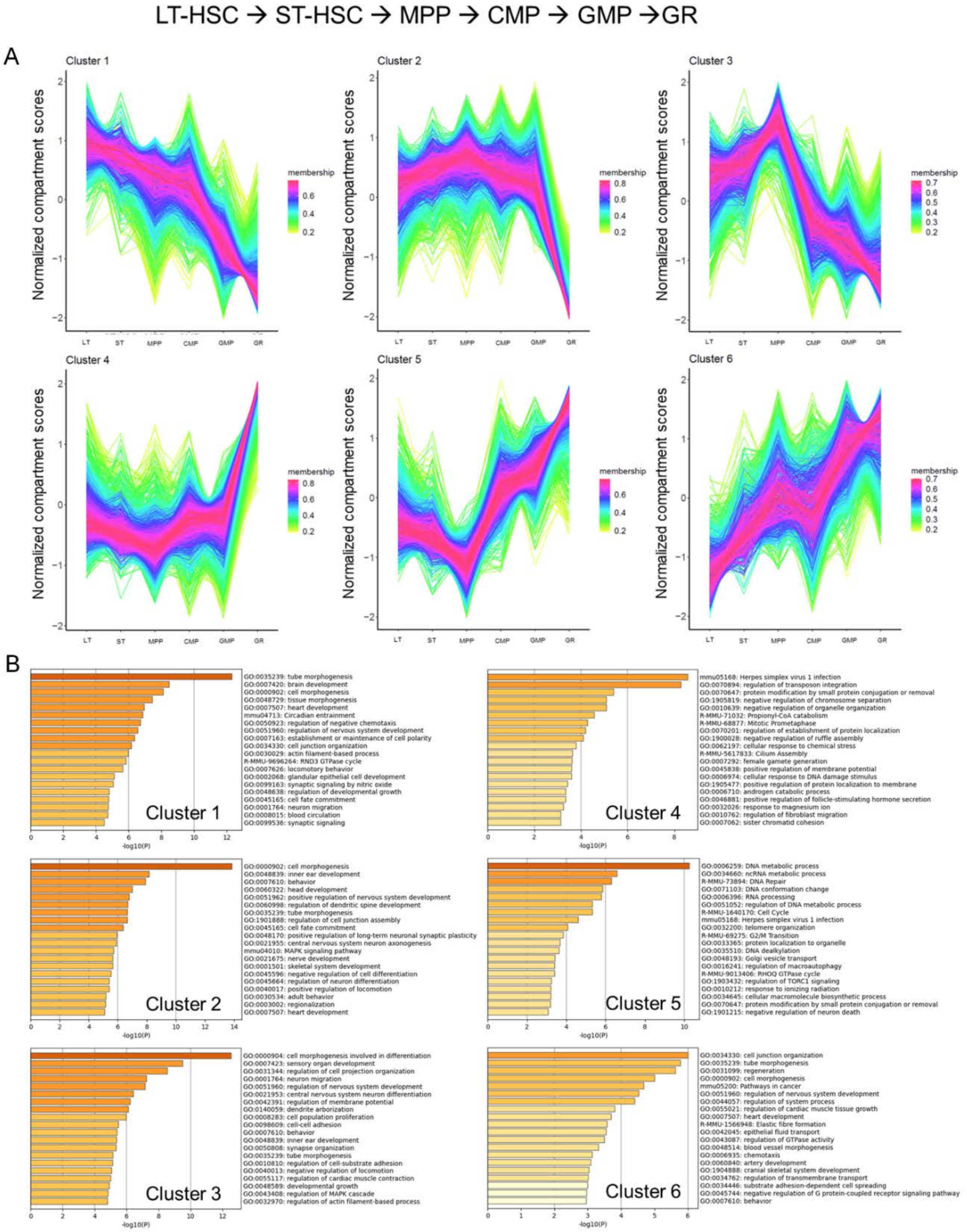

Supplementary Figure S9: Time-series clustering of mouse Long-term hematopoietic stem-cells (LT-HSC) to Megakaryocyte (MK) differentiation based on differential compartment scores. **(A)** Shows the six cluster patterns obtained. **(B)** Functional enrichment results based on the genes overlapping regions within each cluster. Source data are provided as a Source Data file.

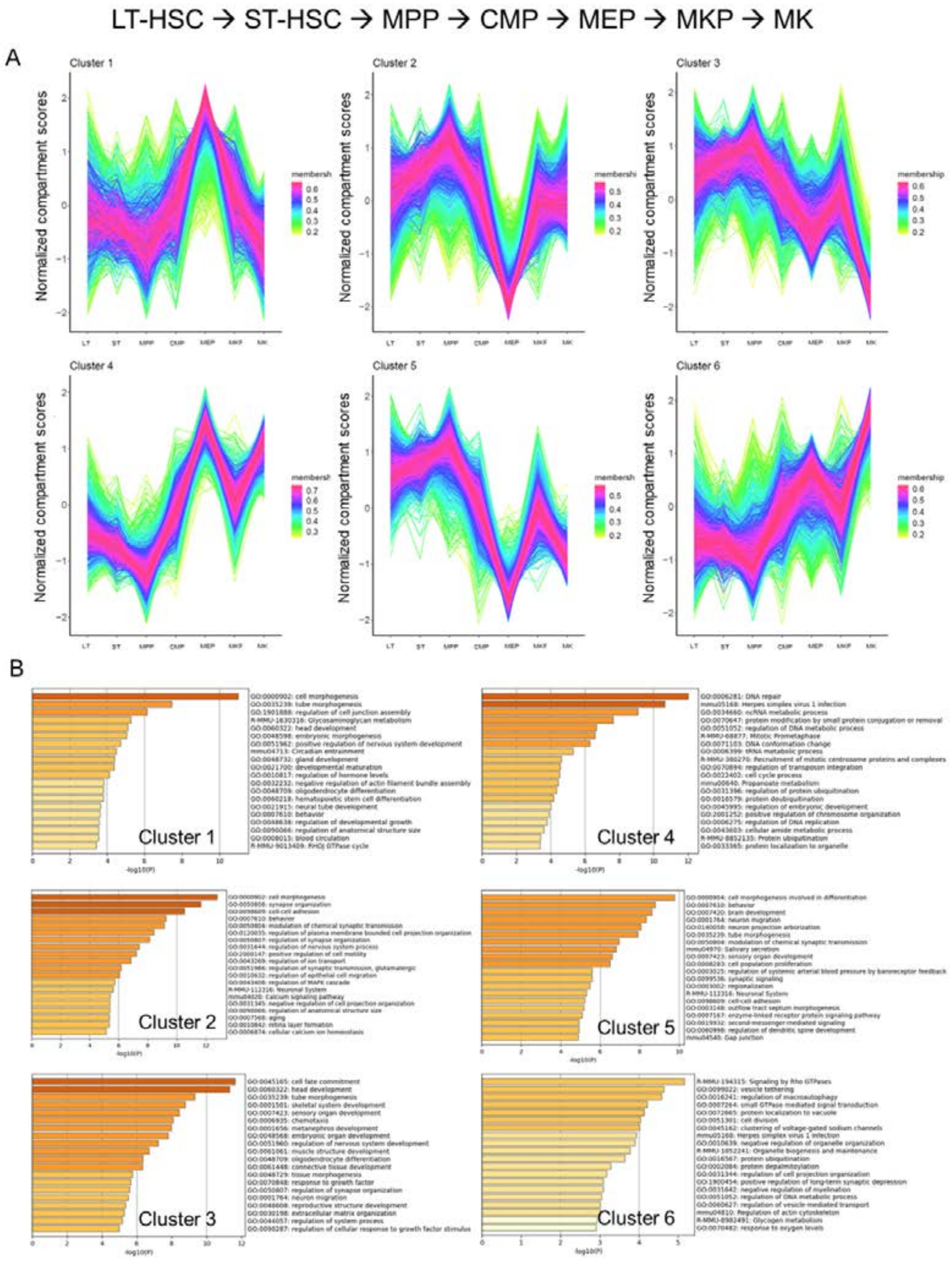

Supplementary Figure S10: Enrichment of differential compartments from either dcHiC or Gorkin et. al. paper that overlap with at least one variable histone mark region/peak identified by Kasowski et. al (2013). The enrichment is computed with respect to all non-differential compartment regions for each method. We assess the enrichment for all calls from dcHiC in comparison to calls that are specific to Gorkin et. al (2019). Source data are provided as a Source Data file.

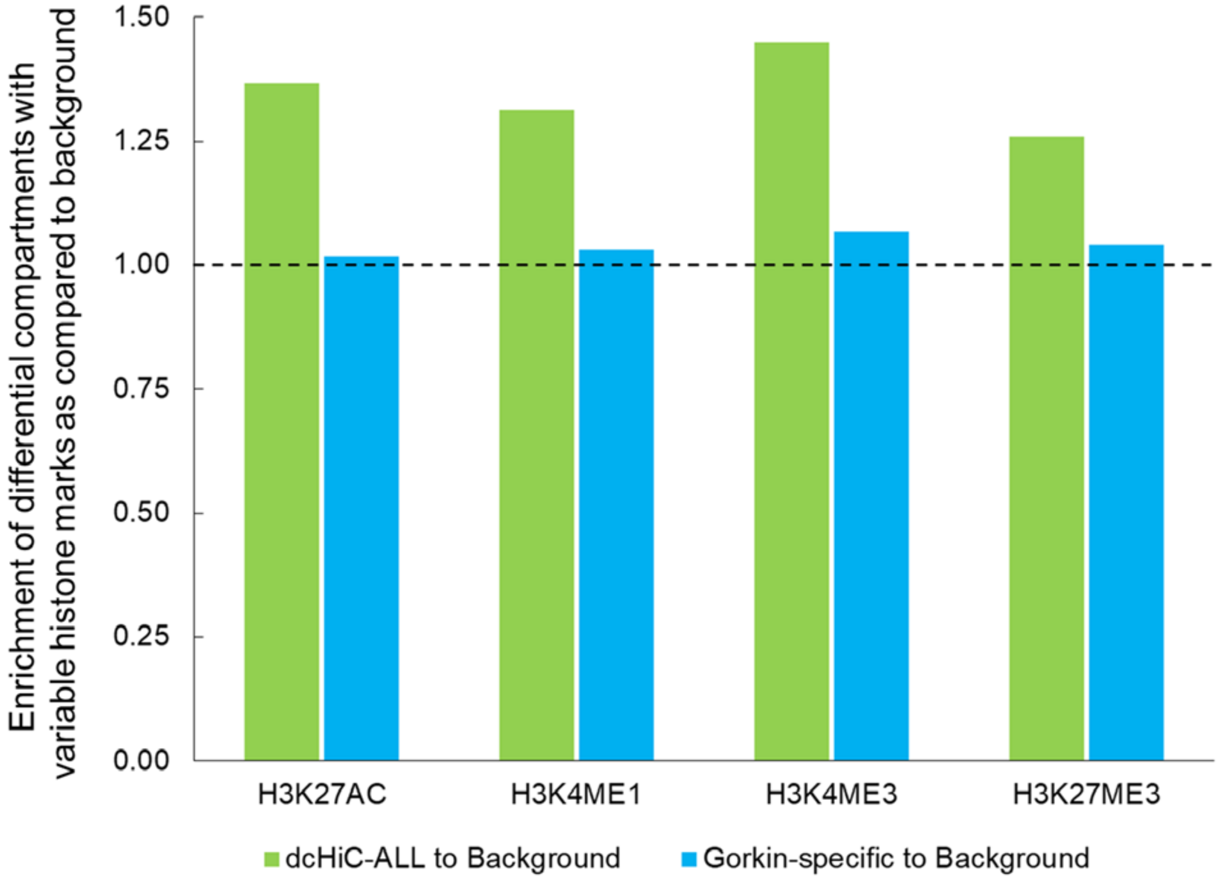

Supplementary Figure S11: The number of differential compartments from each method that overlap with a variable histone mark region (similar to Figure S10) for **(A)** H3K27ac, **(B)** H3K4me3, **(C)** H3K4me1, and **(D)** H3K27me3. Source data are provided as a Source Data file.

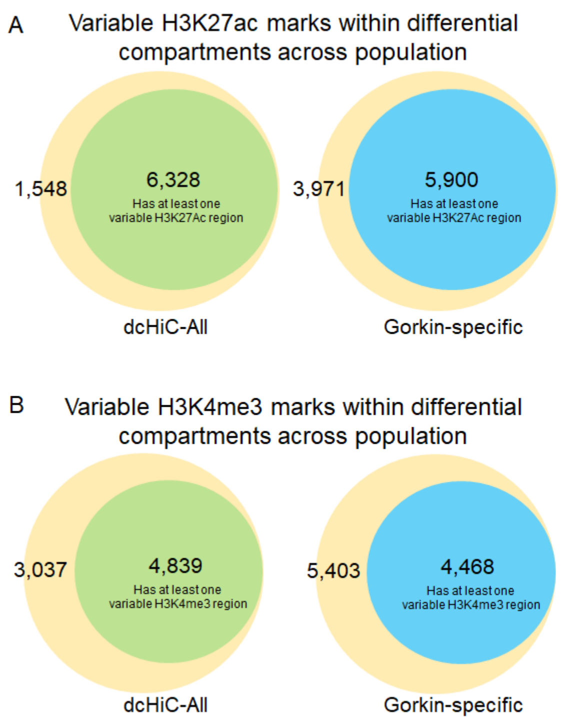

Supplementary Figure S12: Example genes **(A-L)** identified by dcHiC using single-cell 3D Hi-C maps from post-natal mouse brain development data of Tan et. al (2021). The compartment tracks show three stages (early, mid, late; each with two time points combined as replicates) of development for cortex (top) and hippocampus region (bottom). The significance track display -log10(p-values) from dcHiC for each brain region across the three stages. Source data are provided as a Source Data file.

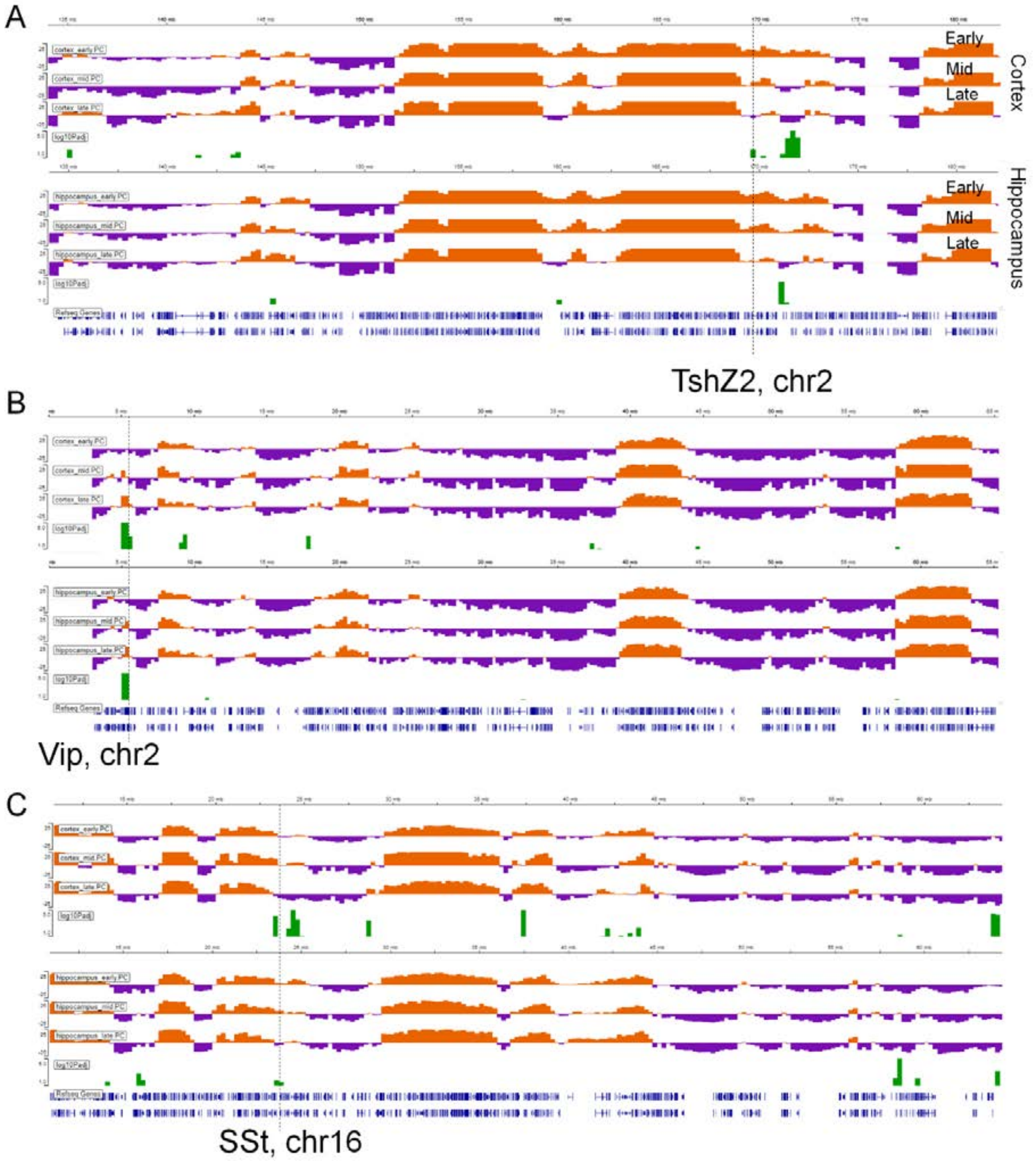

D

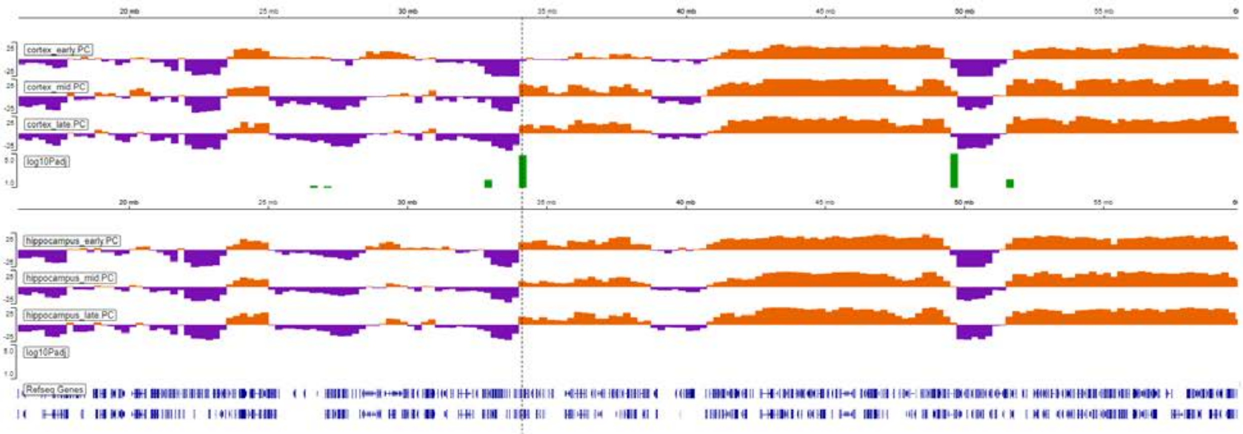

Tubb2b, chr13

E

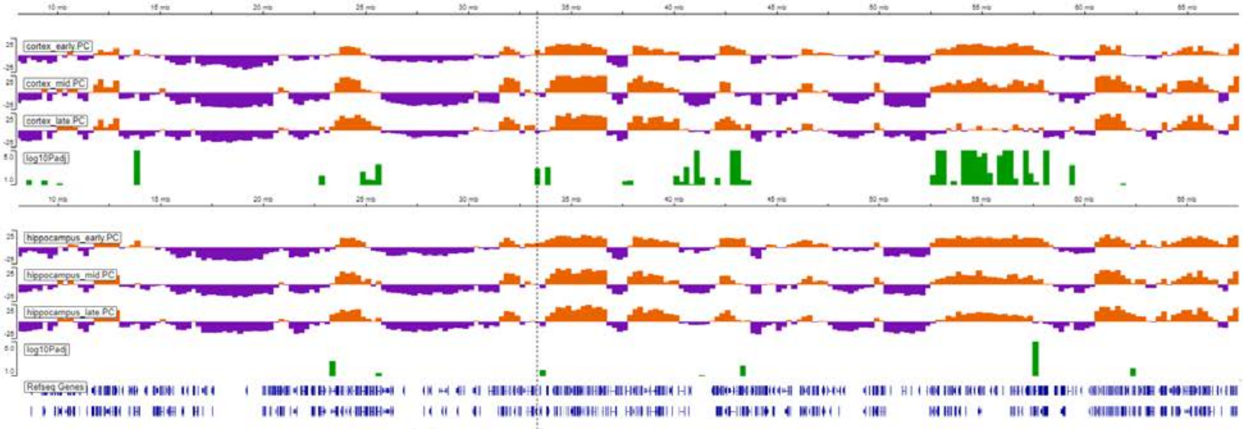

Nrep, chr18

F

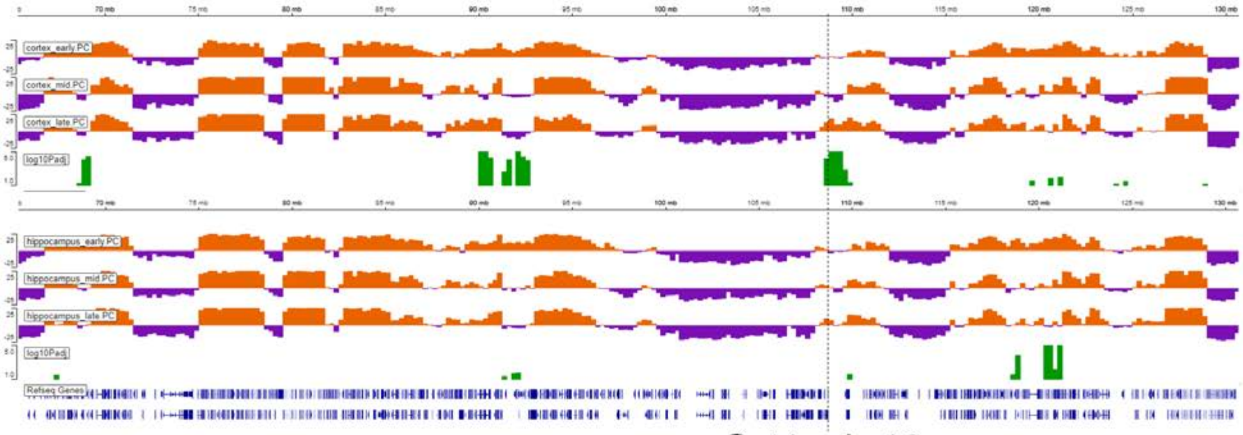

Syt1, chr10

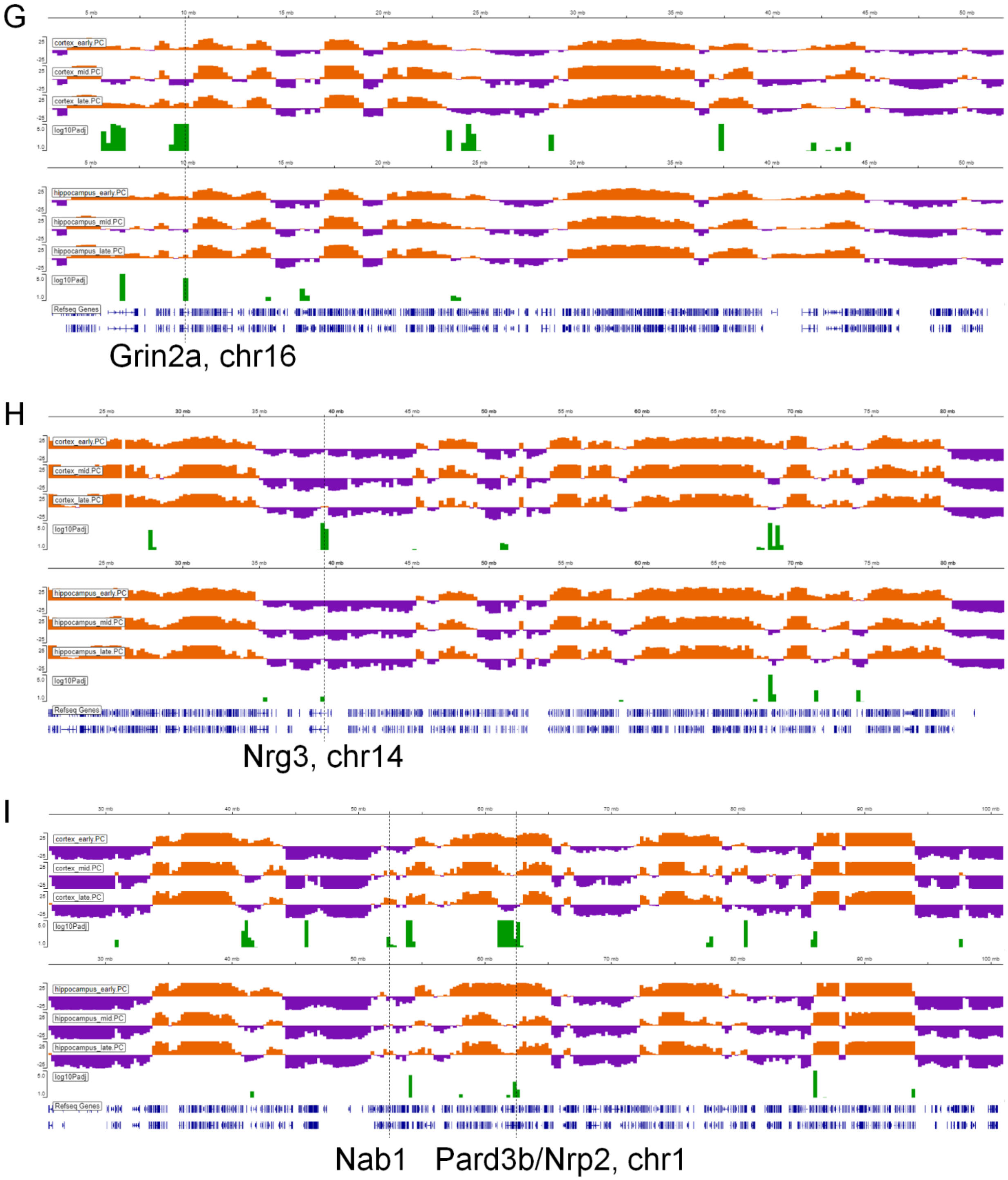
G
cortex_early.PC
cortex_mid.PC
cortex_late.PC
log10Padj
hippocampus_early.PC
hippocampus_mid.PC
hippocampus_late.PC
Refseq Genes
Grin2a, chr16
H
Nrg3, chr14
I
Nab1
Pard3b/Nrp2, chr1

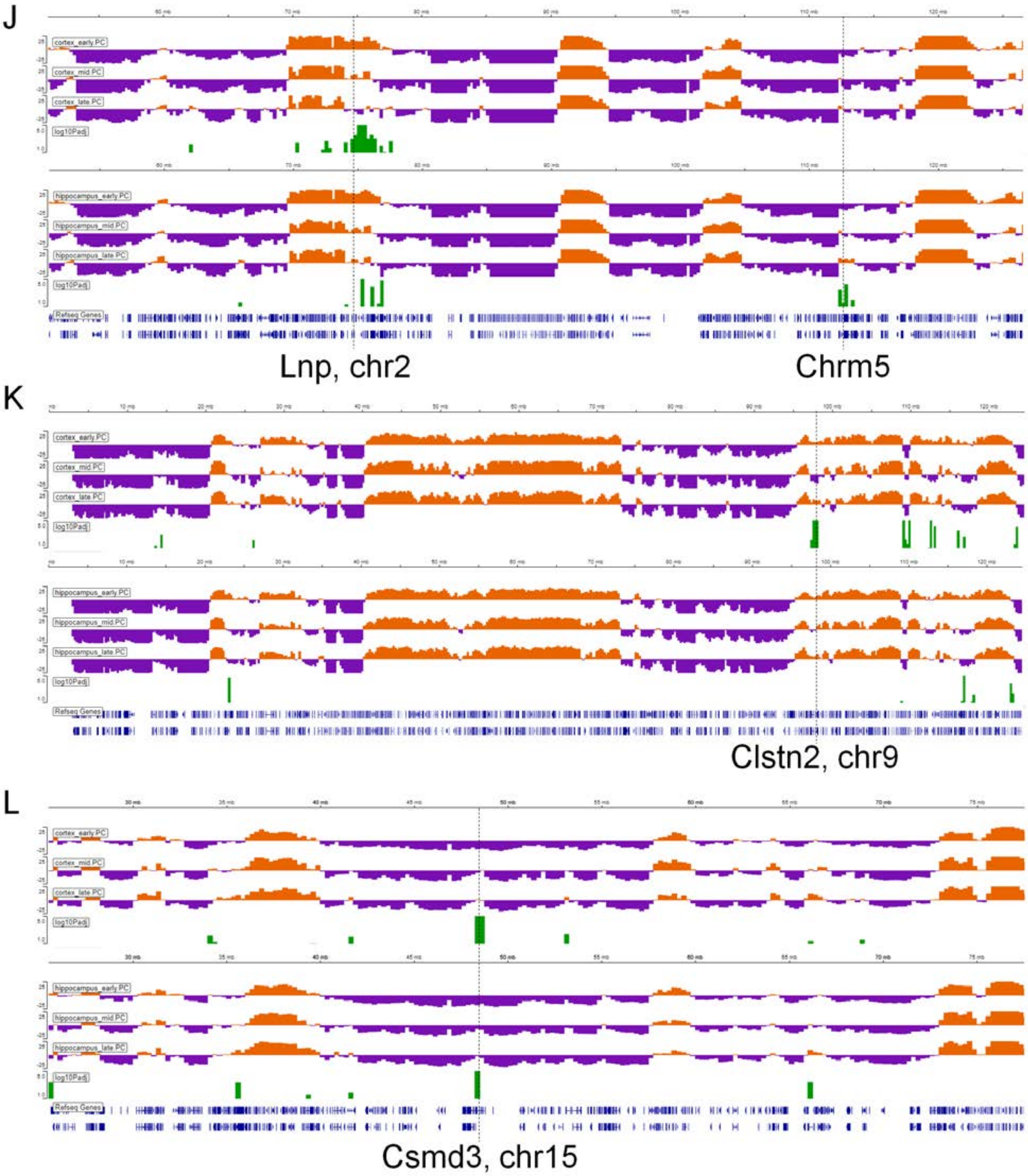
J
cortex_early.PC
cortex_mid.PC
cortex_late.PC
log10Padj
hippocampus_early.PC
hippocampus_mid.PC
hippocampus_late.PC
log10Padj
Refseq Genes
Lnp, chr2
Chrm5
K
cortex_early.PC
cortex_mid.PC
cortex_late.PC
log10Padj
hippocampus_early.PC
hippocampus_mid.PC
hippocampus_late.PC
log10Padj
Refseq Genes
Clstn2, chr9
L
cortex_early.PC
cortex_mid.PC
cortex_late.PC
log10Padj
hippocampus_early.PC
hippocampus_mid.PC
hippocampus_late.PC
log10Padj
Refseq Genes
Csmd3, chr15

Supplementary Figure S13: Time-series analysis of the cortex region during post-natal mouse brain development. **(A-B)** The compartmentalization patters for clusters 3 and 4 of differential compartments. **(C-D)** The functional enrichment categories of genes overlapping with the cluster 3 and 4, respectively. Source data are provided as a Source Data file.

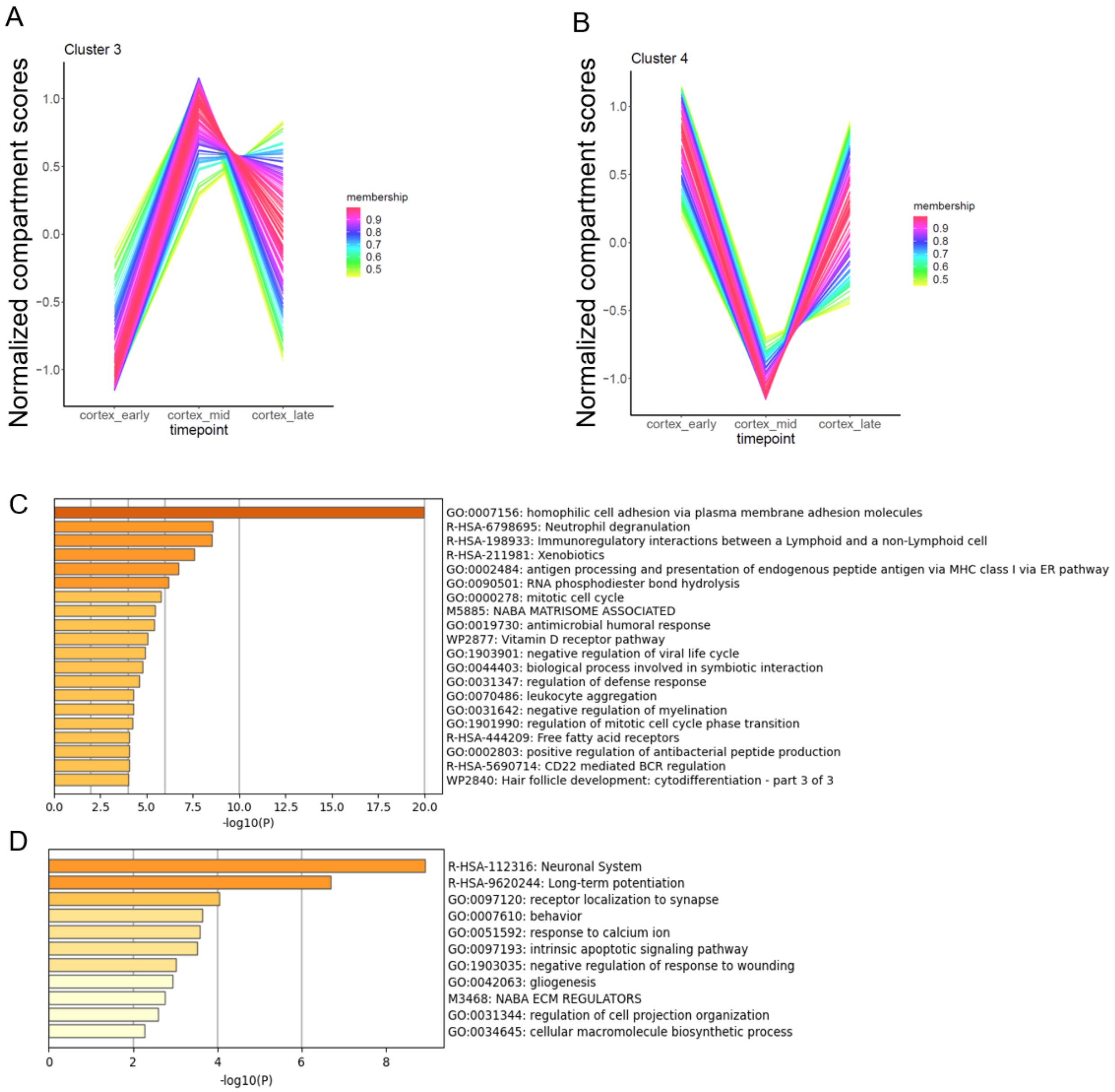

Supplementary Figure S14: Example regions for time-series analysis of the cortex region during post-natal mouse brain development for cluster 3 **(A-B)** and cluster 4 **(C-D)** with their overlapping genes. Source data are provided as a Source Data file.

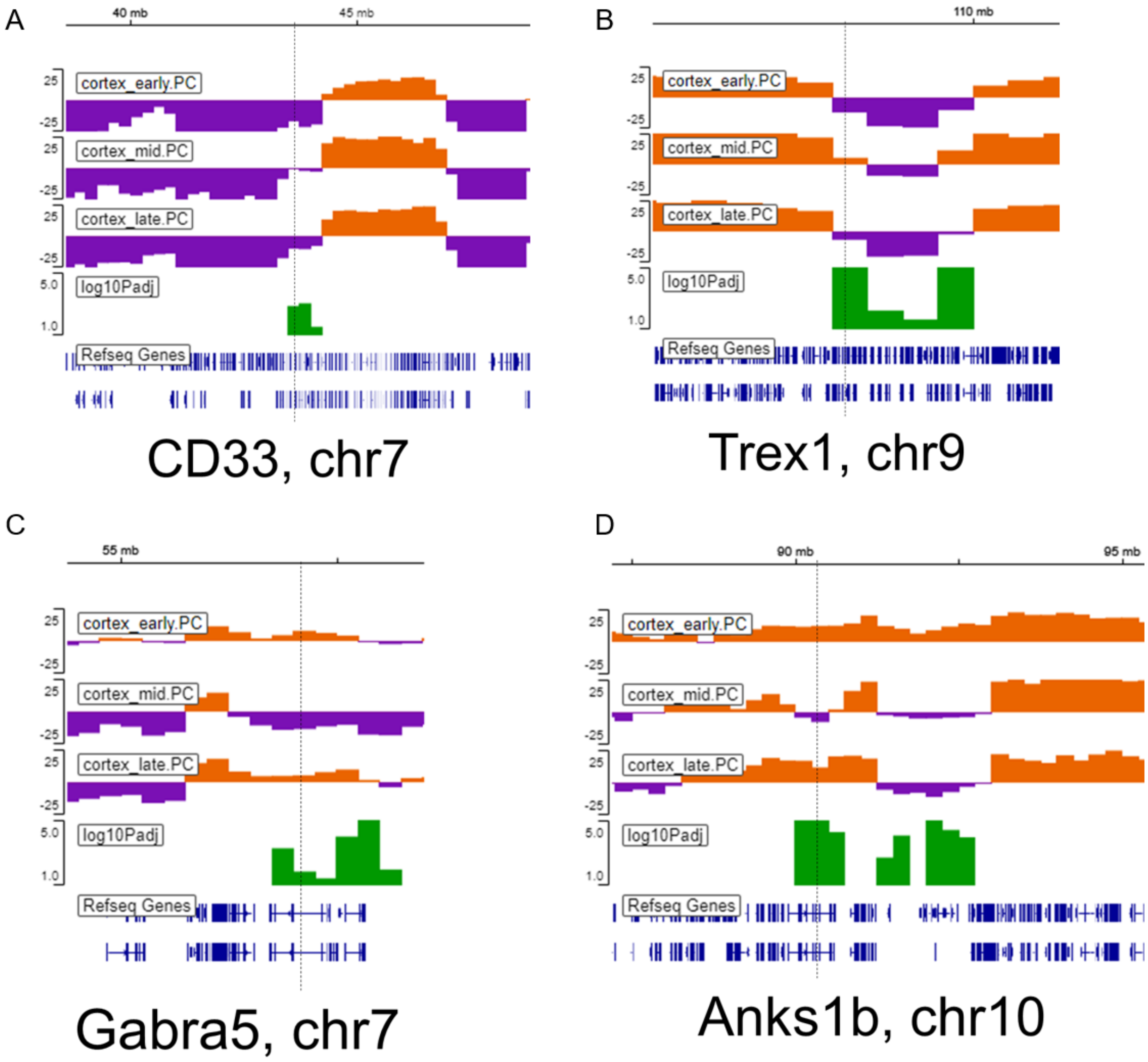

Supplementary Table S10: Publicly available datasets used in this paper.

Accession	Data Type	Cell Lines	Factor(s)	Reference
GSE96107	Hi-C	mESC, NPC, CN	None	Bonev et. al (2017)
GSE96107	RNA-Seq	mESC, NPC, CN	None	Bonev et. al (2017)
GSE96107	ChIP-seq	mESC, NPC, CN	H3K27ac, H3K4me1, H3K4me3	Bonev et. al (2017)
GSE152918	Hi-C	LT-HSC, ST-HSC, MPP, CMP, GMP, GR, MEP, MK	None	Zhang et. al (2020)
GSE128678	Compartment scores	Human population cohort	None	Gorkin et. al (2019)
GSE146397	Single cell 3D contacts	Mouse Cortex and Hippocampus	None	Tan et. al (2021)
GSE50893	ChIP-seq	Lymphoblastoid cell lines (LCLs) derived from 19 individuals	H3K27ac, H3K4me1, H3K4me3, H3K27me3	Kasowski et. al (2013)
