# Supplementary material for: dcHiC detects differential compartments across multiple Hi-C datasets
Source: Nat Commun. 2022 Nov 11;13:6827. doi: 10.1038/s41467-022-34626-6 (PMC9652325; doi:10.1038/s41467-022-34626-6)
Supplement: Supplementary file 3 — Reporting Summary [file 41467_2022_34626_MOESM3_ESM.pdf]

Corresponding author(s): FERHAT AY & ABHIJIT CHAKRABORTY

Last updated by author(s): Oct 26, 2022

Reporting Summary

Nature Portfolio wishes to improve the reproducibility of the work that we publish. This form provides structure for consistency and transparency in reporting. For further information on Nature Portfolio policies, see our Editorial Policies and the Editorial Policy Checklist.

Statistics

For all statistical analyses, confirm that the following items are present in the figure legend, table legend, main text, or Methods section.

n/a	Confirmed	
☐	☒	The exact sample size (*n*) for each experimental group/condition, given as a discrete number and unit of measurement
☒	☐	A statement on whether measurements were taken from distinct samples or whether the same sample was measured repeatedly
☐	☒	The statistical test(s) used AND whether they are one- or two-sided<br>*Only common tests should be described solely by name; describe more complex techniques in the Methods section.*
☐	☒	A description of all covariates tested
☐	☒	A description of any assumptions or corrections, such as tests of normality and adjustment for multiple comparisons
☒	☐	A full description of the statistical parameters including central tendency (e.g. means) or other basic estimates (e.g. regression coefficient) AND variation (e.g. standard deviation) or associated estimates of uncertainty (e.g. confidence intervals)
☐	☒	For null hypothesis testing, the test statistic (e.g. *F*, *t*, *r*) with confidence intervals, effect sizes, degrees of freedom and *P* value noted<br>*Give P values as exact values whenever suitable.*
☒	☐	For Bayesian analysis, information on the choice of priors and Markov chain Monte Carlo settings
☒	☐	For hierarchical and complex designs, identification of the appropriate level for tests and full reporting of outcomes
☒	☐	Estimates of effect sizes (e.g. Cohen's *d*, Pearson's *r*), indicating how they were calculated

Our web collection on statistics for biologists contains articles on many of the points above.

Software and code

Policy information about availability of computer code

Data collection	No specific software was used for data collection
Data analysis	HiCPro (v2.8.0): https://github.com/nservant/HiC-Pro<br>FitHiC2 (v2.0.7): https://github.com/ay-lab/fithic/<br>RNA-seq pipeline (v2): https://github.com/ay-lab/LJI_RNA_SEQ_PIPELINE_V2.git<br>Bowtie2 (v2.1.0): http://bowtie-bio.sourceforge.net/bowtie2/index.shtml<br>MACS (v2.1.1): https://github.com/macs3-project/MACS<br>TC-seq (v1.20.0): https://bioconductor.org/packages/release/bioc/html/TCseq.html<br>IGV Browser (v2.8.0): https://igv.org/<br>bigstatr package (v1.5.12): https://cran.r-project.org/web/packages/bigstatsr/index.html<br>robust package (v0.7-1): https://cran.r-project.org/web/packages/robust/index.html<br>limma (v3.30.13): https://bioconductor.org/packages/release/bioc/html/limma.html<br>hashmap (v0.2.2): https://cran.r-project.org/src/contrib/Archive/hashmap/hashmap_0.2.2.tar.gz<br>Rcpp (v1.0.6): https://cran.r-project.org/src/contrib/Archive/Rcpp/Rcpp_1.0.6.tar.gz)<br>Optparse (v1.20.0): https://cran.r-project.org/src/contrib/Archive/optparse/optparse_1.2.0.tar.gz<br>data.table (v1.10.4-2): https://cran.r-project.org/src/contrib/Archive/data.table/data.table_1.10.4-3.tar.gz<br>RcppEigen (v0.3.3.9.2): https://cran.r-project.org/web/packages/RcppEigen/index.html

For manuscripts utilizing custom algorithms or software that are central to the research but not yet described in published literature, software must be made available to editors and reviewers. We strongly encourage code deposition in a community repository (e.g. GitHub). See the Nature Portfolio guidelines for submitting code & software for further information.

Data

Policy information about availability of data

All manuscripts must include a data availability statement. This statement should provide the following information, where applicable:

- Accession codes, unique identifiers, or web links for publicly available datasets
- A description of any restrictions on data availability
- For clinical datasets or third party data, please ensure that the statement adheres to our policy

The mouse ESC, NPC and CN Hi-C data used in this study are available in the GEO database under the following accession code GSE96107. The mouse hematopoiesis Hi-C data used in this study are available in the GEO database under the following accession code GSE152918. The single-cell Hi-C data used in this study are available in the GEO database under the following accession code GSE146397. The human LCL Hi-C data used in this study are available in the GEO database under the following accession codes GSE128678 and GSE50893. These are also listed in Supplemental Table S10. All reported compartments for all cell lines, multivariate differential scores, RNA-seq, and ChIP-seq data used in this manuscript can be viewed interactively at ay-lab.github.io/dcHiC. These standalone HTML files employ dcHiC's visualization utility through the IGV browser. Source data are provided with this paper.

hg19 genome was downloaded from UCSC genome browser: https://hgdownload.soe.ucsc.edu/goldenPath/hg19/bigZips/
mm10 genome was downloaded from UCSC genome browser: https://hgdownload.soe.ucsc.edu/goldenPath/mm10/bigZips/

Human research participants

Policy information about studies involving human research participants and Sex and Gender in Research.

Reporting on sex and gender	n/a
Population characteristics	n/a
Recruitment	n/a
Ethics oversight	n/a

Note that full information on the approval of the study protocol must also be provided in the manuscript.

Field-specific reporting

Please select the one below that is the best fit for your research. If you are not sure, read the appropriate sections before making your selection.

- [x] Life sciences
- [ ] Behavioural & social sciences
- [ ] Ecological, evolutionary & environmental sciences

For a reference copy of the document with all sections, see nature.com/documents/nr-reporting-summary-flat.pdf

Life sciences study design

All studies must disclose on these points even when the disclosure is negative.

Sample size	No sample size calculation was performed since only published data is used. We utilized all samples and all replicates available, unless stated otherwise
Data exclusions	No data was excluded from the analysis
Replication	Analyses were conducted using different replicates when available. Reproducibility of our results were extensively characterized by downsampling of replicates, changing the contact map resolutions and by creating pseudoreplicates when needed.
Randomization	Only published data were used, therefore, no randomization was applicable.
Blinding	Only published data were used, therefore, no blinding was possible.

Reporting for specific materials, systems and methods

We require information from authors about some types of materials, experimental systems and methods used in many studies. Here, indicate whether each material, system or method listed is relevant to your study. If you are not sure if a list item applies to your research, read the appropriate section before selecting a response.

Materials & experimental systems

n/a	Involved in the study
☒	☐ Antibodies
☒	☐ Eukaryotic cell lines
☒	☐ Palaeontology and archaeology
☒	☐ Animals and other organisms
☒	☐ Clinical data
☒	☐ Dual use research of concern

Methods

n/a	Involved in the study
☒	☐ ChIP-seq
☒	☐ Flow cytometry
☒	☐ MRI-based neuroimaging
