# Supplementary material for: dcHiC detects differential compartments across multiple Hi-C datasets
Source: Nat Commun. 2022 Nov 11;13:6827. doi: 10.1038/s41467-022-34626-6 (PMC9652325; doi:10.1038/s41467-022-34626-6)
Supplement: Supplementary file 4 — Description of Additional Supplementary Files [file 41467_2022_34626_MOESM4_ESM.pdf]

Title: Supplementary Data 1:

Description: This excel file contains functional enrichment analysis per cell type using genes overlapping A compartments with the highest compartmentalization score for that cell type compared to the other two for the comparison of ESC, NPC and CN cell types.

Title: Supplementary Data 2:

Description: This excel file contains the list of genes that were reported in dcHiC differential compartment regions but were either within the A compartment throughout the ESC-NPC-CN transition or within the B compartment. The computed correlation of their compartment scores and gene expression are reported in each sheet.
